# Effects of online mindfulness-based interventions on the mental health of university students: A systematic review and meta-analysis

**DOI:** 10.3389/fpsyg.2023.1073647

**Published:** 2023-02-08

**Authors:** Xiao-Gang Gong, Le-Peng Wang, Guang Rong, Dao-Ning Zhang, A-Yuan Zhang, Chao Liu

**Affiliations:** ^1^Department of Medicine, College of Special Education, Beijing Union University, Beijing, China; ^2^School of Humanities, Beijing University of Chinese Medicine, Beijing, China; ^3^Department of Education, Faculty of Educational Sciences, University of Helsinki, Helsinki, Finland; ^4^Department of Diagnostics of Traditional Chinese Medicine, School of Traditional Chinese Medicine, Beijing University of Chinese Medicine, Beijing, China; ^5^Department of Primary Education, Teachers College, Beijing Union University, Beijing, China; ^6^College of Education, Capital Normal University, Beijing, China; ^7^School of Journalism and Communication, Huaqiao University, Xiamen, China; ^8^Business Analytics Research Center, Chang Gung University, Taoyuan City, Taiwan

**Keywords:** online, mindfulness, mental health, university, students, meta-analysis, systematic review

## Abstract

**Objectives:**

Mental health problems among university students are a cause of widespread concern. Mindfulness-based interventions (MBIs) delivered online have considerable potential to help university students manage mental health challenges. However, there is no consensus regarding the efficacy of online MBIs. This meta-analysis aims to determine whether MBIs are feasible and effective for improving university students’ mental health.

**Methods:**

Randomised controlled trials (RCTs) in Web of Science, PubMed, Cochrane Library, Embase and the US National Library of Medicine (Clinical Trial Registry) published through August 31, 2022, were searched. Two reviewers selected the trials, conducted a critical appraisal, and extracted the data. Nine RCTs met our inclusion criteria.

**Results:**

This analysis showed that online MBIs were effective in improving depression (standardised mean difference [SMD] = −0.27; 95% confidence interval [CI], −0.48 to −0.07; *P* = 0.008), anxiety (SMD = −0.47; 95% CI, −080 to −0.14; *P* = 0.006), stress (SMD = −0.58; 95% CI, −0.79 to −0.37; *P* < 0.00001), and mindfulness (SMD = 0.71; 95% CI, 0.17 to 1.25; *p* = 0.009) in university students. No significant effect was found on wellbeing (SMD = 0.30; 95% CI, −0.00 to 0.60; *P* = 0.05).

**Conclusion:**

The findings indicated that online MBIs could effectively improve the mental health of university students. Nevertheless, additional rigorously designed RCTs are required.

**Systematic review registration:**

https://inplasy.com/inplasy-2022-9-0099/, identifier INPLASY202290099.

## 1. Introduction

Young people’s mental health has been recognised as a global public health problem for a long time and has received increasing attention (Fusar-Poli et al., 2021). Adolescence and early adulthood are considered the peak life stages for the onset of psychiatric disorders, and three-quarters of adults with diagnosable mental health problems experience symptoms of poor mental health status before age 25 (Solmi et al., 2022). University students are especially vulnerable to the effects of stress and are at high risk for alcohol and drug abuse (Fond et al., 2018; Perino et al., 2022). Studies indicate that one-third of university students experience or are experiencing severe mental health problems (Auerbach et al., 2018) and experience higher levels of depression, anxiety, and distress compared with non-students of the same age (Kovess-Masfety et al., 2016; Lo et al., 2020). This can be explained by the fact that the students have cumbersome coursework, poor interpersonal relationships with their classmates and/or teachers, and study–life imbalance (Bergin and Pakenham, 2016; Urbina-Garcia, 2020). These stressors affect their physical and emotional health and lead to declining academic performance, poor life satisfaction, decreased self-confidence, increased dropout rates and, in some cases, suicidal thoughts (Cuijpers et al., 2019; Sheldon et al., 2021). In particular, over the past 3 years, the COVID-19 pandemic has led to serious disturbances to college students’ lives and education, owing to the prolonged closing of educational institutions or delayed school opening, as well as isolation from classmates during the lockdown period. There is evidence that the pandemic dramatically impacted college students’ mental health, with a significant increase in the incidence of psychiatric symptoms during successive lockdowns (Fu et al., 2021; Taylor et al., 2022). In fact, depression, anxiety and stress are still the most common mental problems in the university population (Hamaideh et al., 2022). Therefore, promoting mental health and preventing these mental disorders among university students is paramount.

In recent years, growing evidence suggests that mindfulness-based interventions (MBIs) are becoming increasingly popular among universities, and many apply different types of MBIs to help handle university students’ mental health (Smit and Stavrulaki, 2021; Taylor et al., 2022). Mindfulness can be characterised as the capacity to realise feelings, thoughts, and bodily sensations in the current moment, to have an open and accepted mind toward one’s experience, to understand one’s emotions, and to foster wisdom and love (Sala et al., 2020). In Kabat-Zinn (1982), for the first time, applied mindfulness derived from Buddhist ideas to clinical practice for the treatment of chronic pain. Since then, MBIs have been continuously developed and have been incorporated into various therapies in mental health care, including mindfulness based cognitive therapy (MBCT), mindfulness based stress reduction (MBSR), acceptance and commitment therapy (ACT), and dialectical behavioural therapy (DBT) (Zhang et al., 2021). Among them, the most frequently adopted MBI programmes are MBSR and MBCT (Zhang et al., 2021), and these two types of MBIs have proven effective in reducing some common mental health problems (e.g., anxiety, depression, distress) and physical health problems (Spijkerman et al., 2016; Malboeuf-Hurtubise et al., 2021).

With the rapid development of information technology, increasing numbers of MBIs, including other psychotherapy interventions, are being provided online (Mrazek et al., 2019). Compared with face-to-face interventions, online interventions have many advantages, including: (1) participants can practice in their own space, especially during the COVID-19 pandemic, and can conveniently continue exercising at home; (2) 24-h availability, there is no long waiting list and is easy to join; (3) participants can remain anonymous; and (4) lower cost. In addition, a cross-sectional United States survey showed that, of 500 adults, 42% of the participants preferred individual and online MBIs over group forms (Wahbeh et al., 2014). This indicates that online MBIs can be applied as an alternative to face-to-face interventions. Studies have confirmed the effectiveness of face-to-face MBIs in healthy people and patients with chronic diseases (Zhang et al., 2021). Online MBIS is also considered an effective therapeutic intervention for common psychological problems (Ma et al., 2018; Ungar et al., 2022).

Despite increasing randomised controlled trials (RCTs) studies proving that online MBIs benefit university students’ mental health (Simonsson et al., 2021; Sun et al., 2022), evidence remains weak and sometimes inconsistent.

The impact of online MBIs on psychological problems among university students remains to be comprehensively evaluated in a meta-analysis review. Some earlier meta-analyses of RCTs (Spijkerman et al., 2016; Liu et al., 2022) have evaluated the effects of online MBIs for enhancing mental health aspects (such as depression, anxiety, and stress). However, they primarily concentrated on people with physical conditions, with only a small portion focussing on university students. Although a recent meta-analysis (Dawson et al., 2020) evaluated the impact of MBIs on university students’ mental health, it pools studies that investigated face-to-face mindfulness interventions rather than an online one. To the best of our knowledge, the only study that examined online MBIs for improving the mental health of medical students was conducted and reported in a narrative style (Yogeswaran and El Morr, 2021). Thus, there remains a lack of quantitative evidence regarding the effectiveness of widely used online mindfulness programmes on university students’ mental health, which compelled us to perform this systematic review.

To address this gap in the literature, we formulated three research questions: (1) Are online MBIs effective for improving university students’ mental health compared with active and passive control conditions? (2) What is the effectiveness of online MBIs on mental health in university student populations? (3) What intervention characteristics are common to effective interventions delivered *via* the internet?

Therefore, the main objectives of the current study are threefold. First, we aimed to investigate the evidence for the effectiveness of online MBIs on university students’ mental health and to propose whether to conduct online MBIs for university students in the future. Second, to statistically summarise the reported efficacy of online MBIs on depression, anxiety, and stress. Other factors, such as wellbeing and state of mindfulness, were examined as secondary outcomes. Third, we sought to determine the quality of this evidence. Finally, given the diversity of this population, it is crucial to understand the varying characteristics of the studied population.

## 2. Materials and methods

### 2.1. Study registration

This systematic review and meta-analysis protocol was registered on the International Platform of Registered Systematic Review and Meta-Analysis Protocols (INPLASY) with a registration number of INPLASY 202290099. This study was designed and conducted according to the Preferred Reporting Items for Systematic Reviews and Meta-Analyses (PRISMA) guidelines (Page et al., 2021).

### 2.2. Search strategy

A systematic search for eligible reports of trials was conducted in five online databases: PubMed, Embase, Web of Science, Cochrane Library, and Clinical Trials.gov. All searches ended before August 31, 2022. We used the following main keywords in the initial search: “mindfulness,” “online,” “students,” and “randomised controlled trial.” Afterward, medical subject headings (MeSH) and thesaurus terms were added to construct the specific search terms. The full search string was provided in the Supplementary Appendix.

### 2.3. Inclusion criteria and study selection

We adopted the Populations, Interventions, Comparisons, Outcomes, and Study framework. Studies included in this review were required to meet the following criteria: (1) the study was an RCT; (2) it was conducted using a university student sample; (3) it included an online and MBIs; (4) it included a measurement of mental health outcomes (stress, anxiety, depression, mindfulness state, and wellbeing); and (5) it was available in English.

Studies were excluded if: (1) the intervention was not delivered online (e.g., face-to-face); (2) mindfulness did not form most of the intervention (e.g., yoga, Baduanjin, and ACT); and (3) MBIs were combined with other interventions, such that the individual effects of MBIs could not be assessed.

After removing duplicate articles, all retrieved records were reviewed by two reviewers (X-GG and L-PW) independently. The titles and abstracts of these articles were read to determine whether it was required to retrieve the full text. If either of the reviewers deemed the article inconclusive and required further consideration, they retrieved the full text for review. Subsequently, articles were selected independently by the two reviewers based on the inclusion/exclusion criteria. Any disagreement was resolved by discussing with the corresponding author to reach a consensus.

### 2.4. Appraisal of methodical quality

Two reviewers (X-GG and L-PW) independently assessed the quality of the included RCTs by the Cochrane risk of bias tool (Higgins and Green, 2011). The evaluation contents comprise (1) random sequence generation; (2) allocation concealment; (3) blinding of participants and investigators; (4) blindness of outcome assessments; (5) incomplete outcome data; (6) selective outcome reporting; and (7) other biases. According to the Cochrane Handbook, each domain of the included studies was rated as having a low-risk bias, a high-risk bias, or an unclear risk of bias. Any disagreements were resolved by consensus on the opinion of a third reviewer (A-YZ).

### 2.5. Extraction of the data

The data was extracted in a standardised form by one reviewer (X-GG), and a second reviewer (GR) subsequently tested whether the data was accurate. For the included studies, the following information was extracted: general information (title, authors, year of publication, and geographical location of trials conducted); study characteristics, including baseline sample size, age, gender (% of women), and the number of participants in each group, intervention characteristics (online BMIs programmes, delivery mode, guidance, therapeutic duration, length of session and control group, assessment times), and outcome measures for depression, anxiety, stress (primary outcomes), wellbeing, and mindfulness state (secondary outcome).

For articles with missing data, we contacted the corresponding author through e-mail to request the necessary information. Necessary discussions and consensus with the corresponding author were conducted to settle the disagreements.

### 2.6. Data synthesis

The RevMan 5.4 software of Cochrane Collaboration was used to conduct the meta-analysis (Higgins and Green, 2011). To investigate the effect of online MBIs, separate qualitative analyses were performed on the five different mental health outcomes: depression, anxiety, stress, wellbeing, and mindfulness. Their effect sizes were summarised using the inverse variance of the individual studies as weights. For each outcome, quantitative data was provided, and the weighted mean difference (WMD) with its 95% confidence interval (CI) was reported. The statistical heterogeneity of included studies was assessed by *I*^2^ statistics. *I*^2^ values between 25 and 50%, 25 and 50%, and >50%, respectively, indicated mild, moderate, and high heterogeneity. A fixed-effects model was used with an *I*^2^ < 50% and *p* > 0.1. Otherwise, we switched to a random-effects model. When study data could not be collected, a narrative synthesis was done. When at least ten publications were included, funnel plots were used to detect potential publication bias.

## 3. Results

### 3.1. Literature search

**FIGURE 1:**
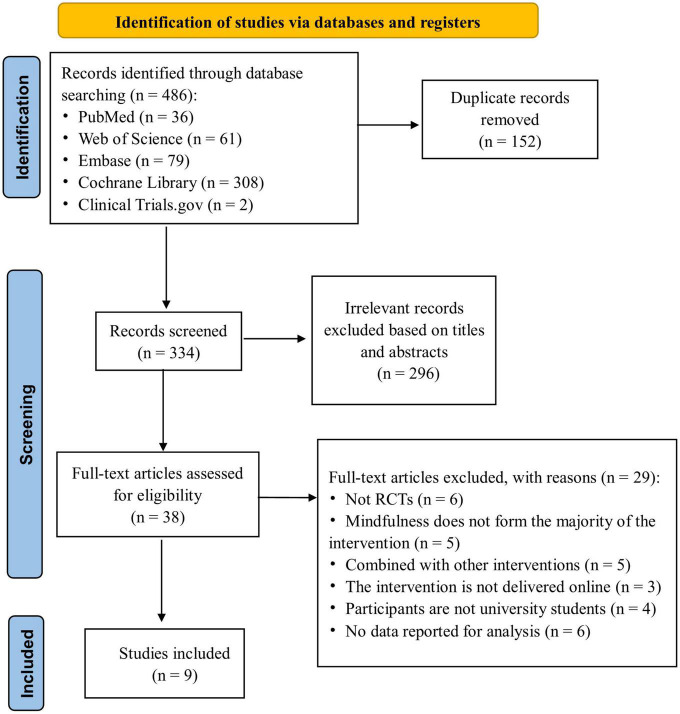
Flow chart of study selection.

A total of 486 potential studies were identified in the initial search for the analysis. After removing 152 duplicated articles, 334 studies remained, of which 296 were excluded after the screening of titles and abstracts. We reviewed the remaining 38 full-text articles and excluded 29 studies that did not meet the inclusion criteria: (1) no RCTs (*n* = 6), (2) mindfulness did not form the majority of the intervention (*n* = 5), (3) combined with other interventions (*n* = 5), (4) the intervention was not delivered online (*n* = 3), (5) participants were not university students (*n* = 4), and (6) no relevant data reported for analysis (*n* = 6). Finally, nine RCTs were included in this review (Cavanagh et al., 2013; Noone and Hogan, 2018; Yang et al., 2018; Huberty et al., 2019; Throuvala et al., 2020; Orosa-Duarte et al., 2021; Simonsson et al., 2021; Kam et al., 2022; Sun et al., 2022). The detailed process of the study selection is illustrated in Figure 1.

### 3.2. Study characteristics

Table 1 displays the summarised characteristics of the included studies. Overall, nine RCTs were conducted in six countries: the UK (*n* = 3) (Cavanagh et al., 2013; Throuvala et al., 2020), USA (*n* = 2) (Yang et al., 2018; Huberty et al., 2019), Canada (*n* = 1) (Kam et al., 2022), Ireland (*n* = 1) (Noone and Hogan, 2018), Spain (*n* = 1) (Orosa-Duarte et al., 2021) and China (*n* = 1) (Sun et al., 2022). These studies yielded 1,100 participants (all university students; mean age = 20–30 years), of which 557 received MBIs, and 543 were included in the control sample. One study reported that the participants were students of psychology background (Throuvala et al., 2020), one reported the students were from a medical university (Yang et al., 2018), and the remaining RCTs did not mention the background of student participants. The study sample size ranged from 62 to 252. Women covered over six percent of the total participants in all studies. Six studies examined MBCT, one MBSR, one MBSH, and one applied a combination of MBCT and MBSR interventions. Three studies performed interventions with guidance, while others performed without guidance. According to delivery mode, the experimental groups can be divided as follows: mobile applications (*n* = 6), videoconferences (*n* = 1), and websites (*n* = 2). The duration of online MBIs in the intervention groups varied from 10 days to 2 months, and the training sessions varied from 4 to 14. Seven RCTs used an inactive control condition, all of which were waiting list groups. An active control was used in the remaining two RCTs, in which participants got social support (*n* = 1) or sham meditation (*n* = 1). The primary outcome measures were depression in four comparisons, anxiety in six comparisons, and stress in four comparisons. Secondary outcome measures were wellbeing in two comparisons and mindfulness in six comparisons. All the instruments possess good psychometric natures. After the intervention, follow-up periods of 1 to 3 months were investigated in the three studies. No adverse events related to online MBIs were reported in the included RCTs.

**TABLE 1:** Characteristics of included studies.

References	Study location	BSZ (MBIs/CG)	%F^a^	Age, mean (SD) (years)	Intervention	Guidance	Deliverymode	N sessions, duration in weeks	Control group	Measurements	Outcome measured
Cavanagh et al., 2013	United Kingdom	104 (54/50)	88.5	24.7(6.4)	MBSH	Without	Website	14 sessions, 2 weeks	Inactive (waitlist)	Pre, post	Depression/anxiety: PHQ-4; stress: PSS; mindfulness: FFMQ
Huberty et al., 2019	United States	109 (56/53)	88.9	21.18 (5.5)	MBCT	Without	Mobile application	8 sessions, 8 weeks	Inactive (waitlist)	Pre, post, 12-weeks follow-up	Stress: PSS; mindfulness: FFMQ
Kam et al., 2022	Canada	62 (32/30)	83.9	29.9 (8.8)	MBCT	Without	Mobile application	10 sessions, 2 weeks	Inactive (waitlist)	Pre, post	Depression/anxiety: PROMIS
Noone and Hogan, 2018	Ireland	91 (48/43)	75.8	20.92 (4.4)	MBCT	With	Website	6 sessions, 6 weeks	Active (sham meditation)	Pre, post	Wellbeing: WEMWS; mindfulness: FFMQ
Orosa-Duarte et al., 2021	Spain	103 (54/49)	84.5	23 (4.2))	MBSR	Without	Mobile application	8 sessions, 8 weeks	Inactive (waitlist)	Pre, post	Anxiety: STAI-T; mindfulness: FFMQ
Simonsson et al., 2021	United Kingdom	177 (88/89)	64.4	23.27 (5.6)	MBCT	With	Videoconferencing	8 sessions, 8 weeks	Inactive (waitlist)	Pre, post, 1-month follow-up	Depression/anxiety: PROMIS
Sun et al., 2022	China	114 (57/57)	73.7	22.21 (2.7)	MBSR and MBCT	With	Mobile application	4 sessions, 4 weeks	Active (social support)	Pre, post, 2-months follow-up	Depression: PHQ-9 anxiety: GAD-7 mindfulness: MAAS
Throuvala et al., 2020	United Kingdom	252 (123/129)	82	20.72 (3.1)	MBCT	Without	Mobile application	10 sessions, 10 days	Inactive (waitlist)	Pre, post	Anxiety: GAD-7 stress: PSS; mindfulness: MAAS
Yang et al., 2018	United States	88 (45/43)	63.6	25.11	MBCT	Without	Mobile application	4 sessions, 2 months	Inactive (waitlist)	Pre, post	Stress: PSS; wellbeing: GWBS; mindfulness: FFMQ

BSZ, baseline sample size; CG, control group; FFMQ, five facets of mindfulness questionnaire; GAD-7, generalised anxiety disorder-7; GWBS, general wellbeing schedule; MAAS, mindful attention awareness scale; MBCT, mindfulness-based cognitive therapy; MBIs, mindfulness-based interventions; MBSH, mindfulness-based self-help; MBSR, mindfulness-based stress reduction; PHQ-4, patient health questionnaire for depression and anxiety; PHQ-9, patient health questionnaire; PROMIS, patient-reported outcomes measurement information system; PSS, perceived stress scale; STAI-T, state-trait anxiety inventory–trait subscale; UK, United Kingdom; USA, United States; WEMWS, Warwick-Edinburgh mental wellbeing Scale; %F^a^, percentage of women in the total study population at baseline.

### 3.3. Methodological quality

**FIGURE 2:**
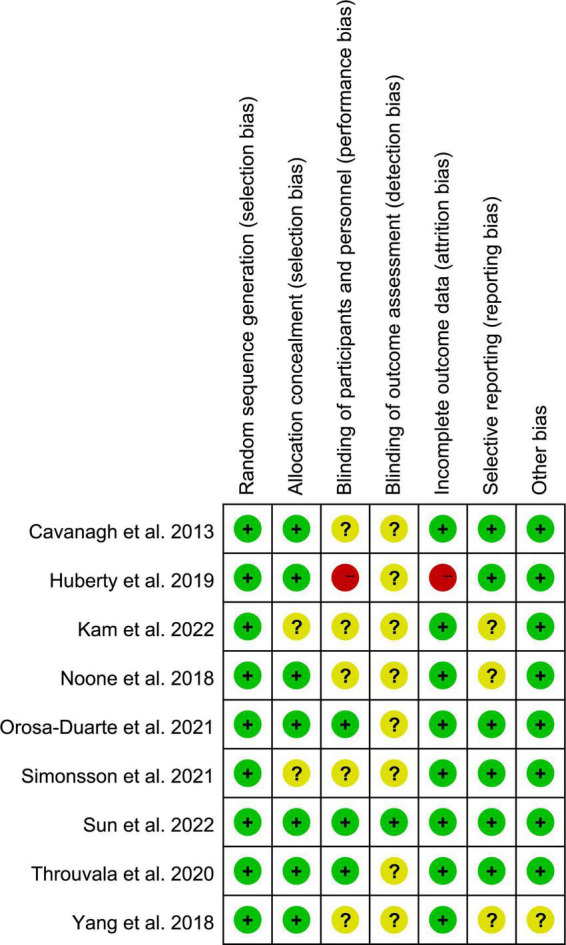
Summary of bias risk for included studies.

The risk of bias assessment for each included study is summarised in Figure 2. Random sequence generations were applied in all studies. Seven studies described specific methods of allocation concealment, which were not provided by the remaining two studies. The outcome assessors were blinded in two trials, and the remaining RCTs rated as unclear for detection bias were not reported. The participants and/or personnel were blinded in three studies; one study reported that blinding was not used. One study reported partial data loss and was considered at a high risk of attrition bias; the remaining RCTs were rated as low risk. Six RCTs reported trial registration rated as low risk, and the remaining three, with no mention, were rated as unclear selective reporting bias. Only one RCT did not report that the authors received a government grant, so the trial was rated as having an unclear other bias.

### 3.4. Meta-analysis outcome

#### 3.4.1. Primary outcomes

##### 3.4.1.1. Effects on depression

**FIGURE 3:**
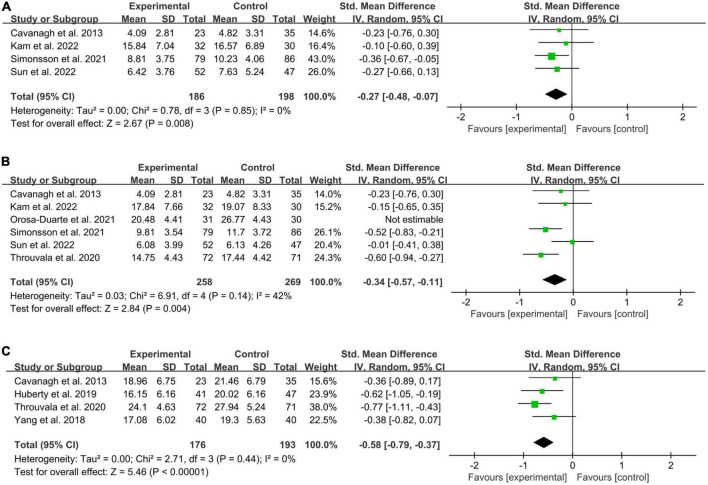
Forest plot of meta-analysis for the efficacy of online MBIs on mental health. **(A)** Depression. **(B)** Anxiety. **(C)** Stress.

Four included studies (Cavanagh et al., 2013; Simonsson et al., 2021; Kam et al., 2022; Sun et al., 2022) involving 186 online MBIs and 198 control subjects assessed depression outcomes (Figure 3A). The results were *I*^2^ = 0% and *p* = 0.85, indicating that heterogeneity was negligible. Compared with the control condition, the meta-analysis found a significant effect of online MBIs in alleviating depression (SMD = −0.27; 95% CI, −0.48, −0.07; *P* = 0.008).

##### 3.4.1.2. Effects on anxiety

Six included studies (Cavanagh et al., 2013; Throuvala et al., 2020; Orosa-Duarte et al., 2021; Simonsson et al., 2021; Kam et al., 2022; Sun et al., 2022) involving 289 participants in the online MBIs intervention groups and 299 controls were analysed to determine the effect on anxiety (Figure 3B). A high level of heterogeneity was observed (*P* = 0.002; *I*^2^ = 73%). For the meta-analysis, compared with controls, aggregated results showed significant benefit in favour of online MBIs on anxiety (SMD = −0.47; 95% CI, −080 to −0.14; *P* = 0.006). By examining the forest plot, a potential outlier was identified (Orosa-Duarte et al., 2021). After removing outliers, the pooled effect was reduced to SMD = 0.34, 95% CI (0.57, 0.11). However, we found that the effect of improving anxiety remained significant (*P* = 0.004). In addition, the heterogeneity was reduced to a moderate level (*I*^2^ = 42%, *P* = 0.14).

##### 3.4.1.3. Effects on stress

We successfully included four studies (Cavanagh et al., 2013; Yang et al., 2018; Huberty et al., 2019; Throuvala et al., 2020) involving 176 online MBIs and 193 control participants (Figure 3C). The results showed that online MBIs were more effective than the controls in alleviating stress (SMD = −0.58; 95% CI, −0.79 to −0.37; *P* < 0.00001). No significant heterogeneity was found between studies (*P* = 0.44; *I*^2^ = 0%).

#### 3.4.2. Secondary outcomes

##### 3.4.2.1. Effects on wellbeing

**FIGURE 4:**
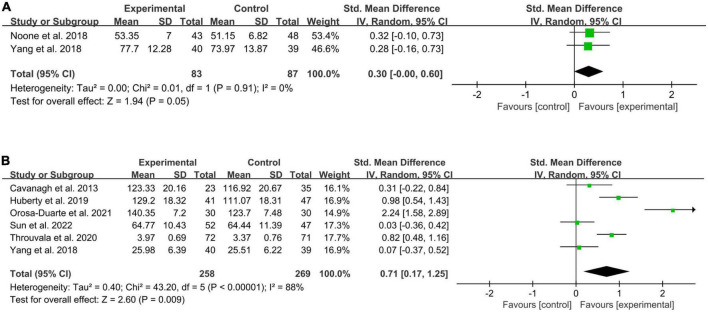
Forests plot of meta-analysis for the effect of online MBIs on wellbeing and mindfulness. **(A)** Wellbeing. **(B)** Mindfulness.

Of the nine RCTs, only two (Noone and Hogan, 2018; Yang et al., 2018) reported the data on wellbeing (Figure 4A). The results showed *I*^2^ = 0% and *p* = 0.91, exhibiting no statistical heterogeneity. Compared with the control condition, the meta-analysis showed no statistically significant improvement in wellbeing in the online MBIs group (SMD = 0.30; 95% CI, −0.00 to 0.60; *P* = 0.05).

##### 3.4.2.2. Effects on mindfulness

Six RCTs (Cavanagh et al., 2013; Yang et al., 2018; Huberty et al., 2019; Throuvala et al., 2020; Orosa-Duarte et al., 2021; Sun et al., 2022) reported the data on mindfulness (Figure 4B). The meta-analysis indicated that online MBIs had a significant effect on mindfulness (SMD = 0.71; 95% CI, 0.17 to 1.25; *P* = 0.009) compared with the control condition. The results were *I*^2^ = 88% and *P* < 0.00001, indicating high statistical heterogeneity. By assessing the forest plot, a potential outlier was identified (Orosa-Duarte et al., 2021). After removing outliers, the pooled effect was reduced to SMD = 0.45, 95% CI (0.05, 0.84), yet there still was a significant statistical effect (*P* = 0.03). In addition, the level of heterogeneity was high (*I*^2^ = 77%, *P* = 0.002).

## 4. Discussion

### 4.1. Main findings

The present meta-analysis identified nine RCTs with 1,100 participants to examine the effectiveness of online MBIs on university students’ mental health. The pooled analyses demonstrated that online MBIs reduced depression, anxiety, and stress and improved mindfulness significantly. Nevertheless, we did not detect an overt substantial impact on wellbeing.

The fact that eight out of the nine studies were just recently published highlights the growing interest in this kind of intervention. The impact of online MBIs on the mental health of all college student groups remains unproven, despite the rise in studies in this area. Our findings in this study are comparable to those from the earlier publication of MBIs based on a healthy population (Khoury et al., 2015; Liu et al., 2022). Prior meta-analyses did not, however, exclusively focus on university students, and the MBI types examined in these studies also varied. As a result, this meta-analysis is the first to show how online MBIs affect university students’ mental health. Concerns about the mental health of university students have grown, and if temporary academic psychological hurdles are not removed, this could result in long-term mental and physical illnesses, even suicidal tendencies. The study’s findings could provide evidence for an easy-to-use tool that university students could adopt to manage mental health problems, including depression, anxiety, and stress.

We observed that the effect sizes of online MBIs on depression, anxiety, stress, and mindfulness in this meta-analysis were generally larger for university students than those found for all individuals with physical health conditions (including non-university students) in previous research (Liu et al., 2022). This may be related to university students being more familiar with online and mobile applications and having an advantage in practising online MBIs. Eight of the nine included studies in this review used mobile applications or websites as the delivery modes of the experimental groups.

Although online MBIs were shown to increase wellbeing to some level in the reports of two RCTs (Noone and Hogan, 2018; Yang et al., 2018), a meta-analysis of these two trials in this investigation revealed that there was no statistically significant improvement in wellbeing. Different findings from earlier studies have been found regarding this outcome. Online MBIs have been found to considerably impact wellbeing in one study (Sevilla-Llewellyn-Jones et al., 2018), but their impact on quality of life is modest (Spijkerman et al., 2016). These meta-analyses’ contradictory results may be explained by several factors, including how the programme was delivered or samples from different groups and physical health situations (Vollestad et al., 2012). This might be due to the small number of studies that report wellbeing and the significant degree of heterogeneity; as a result, multicenter, high-quality, large-sample research is expected to investigate how online MBIs affect wellbeing.

Additionally, in studies with both a waitlist and an active control group, some outcomes improved more in online MBIs than in these two control groups (Orosa-Duarte et al., 2021), indicating that MBIs are equally effective as or even more effective than other active interventions in alleviating some mental problems.

We still do not fully understand how MBIs affect the human body to enhance mental wellbeing and relieve psychiatric issues. An 8-week mindfulness-based therapy programme resulted in changes in the structure and function of neurons, according to a prior review article (Guendelman et al., 2017). Following MBIs, healthy and unhealthy patients showed enhanced functional activity and structural connectivity in the cingulate cortex, prefrontal cortex, hippocampus, and insula. The amygdala also displayed decreased functional activity and enhanced connection with the prefrontal cortex, resulting in improved emotional control (Gotink et al., 2016). A recent study shows that MBSR reduces the pressure of self-reporting and controls its pain through cognitive reassessment and acceptance. In addition, over time, MBSR enhances brain activity through cognitive reappraisal and acceptance to manage its own (parietal cortex) emotions (Guendelman et al., 2022). These may lead to increased capacity for resilience following MBI.

Online intervention for mental health issues has proliferated and is expected to outperform traditional face-to-face therapies alone in terms of accessibility, acceptance, scalability, and cost-effectiveness (Ferrari et al., 2022). Online therapies aimed at university students’ psychological issues may eliminate their worries about stigma, time constraints, and unfamiliarity with the healthcare system (Montagni et al., 2020). Moreover, the high usage of smartphones and familiarity with blended learning modes mean that most university students are well suited for an online digital health support model (Ferrari et al., 2022). Other global factors, like the COVID-19 pandemic, necessitate social distancing and prolonged in-room hours to slow the spread of the virus. An effective online psychological intervention for university students, therefore, seems especially appropriate and necessary.

A previous systematic review (Zhou et al., 2021), including 45 RCTs with 13,291 participants, indicated that online mental health interventions were effective in managing various mental health conditions (such as depression, anxiety, stress, insomnia, and improving quality of life) among youth when compared with control conditions. The current meta-analysis provides enhanced evidence for the efficacy of online mental health intervention. The result affirms the value of existing theory in this area, and we anticipate that it could contribute to the development of practice-oriented guidelines.

One study compared online and face-to-face mindfulness and discovered that both had certain advantages over the other in different indicators (Orosa-Duarte et al., 2021). Although the authors did not delve further into the mechanism at play, it appears that the flexibility of online mindfulness gives it a competitive edge. Nonetheless, more research is necessary to support this observation.

Additionally, this review includes the most traditional and widely used MBI programmes, such as MBSR and MBCT (Zhang et al., 2021), and these interventions were primarily conducted on a psychological level. According to a broad definition, some traditional Chinese Integrative Body-Mind Training (IBMT) regimens, like Tai Chi, Baduanjin, and Qigong, were also included in the category of MBIs (Creswell, 2017). Research has shown that these exercise regimens can benefit practitioners’ physical and mental health (Zou et al., 2018; Li et al., 2019; Gong et al., 2022; Lin et al., 2022). Researchers should continue to explore what modifications are necessary for these IBMT programmes to be delivered online for university students.

### 4.2. Strengths and limitations of the review

To the best of our knowledge, this is the first meta-analysis to examine the effectiveness of online MBIs designed specifically for university students with mental health issues. We conducted a comprehensive analysis of the evidence of the included RCTs, which are considered to be the most appropriate and recommended method to evaluate the intervention effect (Kabisch et al., 2011). What is more, the total number of samples included in most meta-analyses was relatively sufficient, and these were distributed in a wide range of geography, covering six countries (China, the USA, Canada, Ireland, the UK, and Spain) on three continents (Europe, America, and Asia), which may reinforce the generalisation of research conclusions.

Despite the apparent positive effects of online MBIs on university students’ mental health, a drawback of this framework should be noted.

First, methodological risks or other inadequacies are present in most of the included studies, which limits the strength and feasibility of clinical evidence. One of the most significant drawbacks of most studies is the lack of blinding. Five of the nine studies did not report participant and/or personnel blinding, and one reported that blinding was not used. In addition, only one study reported the adoption of outcome assessment blinding, and one study was considered to have a high risk of attrition bias for reporting partial data loss. Because of these biases, we should be cautious when interpreting the results of this systematic evaluation. Significant heterogeneity in the meta-analysis of this article was found, which may be due to differences in method quality, participants, interventions, and outcome evaluation. Therefore, it is necessary to conduct more rigorous studies with higher standards of trial methodology to assess the effects of online MBIs on the mental health of university students.

Second, in different studies that meet the inclusion criteria, the duration and frequency of online MBIs were significantly different. The duration of the intervention varied from 10 days to 2 months, and the training sessions varied from 4 to 14 times a week, which may have different effects on online MBIs in alleviating anxiety, depression, and stress and improving mindfulness. It was unclear whether the therapeutic effect varied with the intervention length, session, and frequency. Thus, the differences in practice make it difficult to make specific suggestions on the frequency and duration of practice.

Third, our bibliographic search was restricted to English publications. Additionally, we did not search for unpublished data. Both these aspects may have hindered our ability to identify other relevant trials. This meta-analysis was finally based on nine studies, and the small number of eligible RCTs was a limitation. Because it limits the reliability and validity of statistics, it may explain why the changes in some analysis results do not reach statistical significance. We expect that more rigorously designed and large-scale trials can help us address these shortcomings in the future.

## 5. Conclusion

Based on the available studies, this meta-review shows that online MBIs may effectively improve depression, anxiety, stress, and mindfulness state among university students. Although current research exploring the effectiveness of online MBIs is still in the early stages, we conclude that there is emerging evidence that online MBIs have the potential to improve university students’ mental health. In addition, more rigorous RCTs with larger sample sizes are warranted to establish the therapeutic effects of online MBIs on mental health problems (depression, anxiety, and stress) and to improve mindfulness state and wellbeing, particularly among university students.

## Data availability statement

The original contributions presented in this study are included in the article/Supplementary material, further inquiries can be directed to the corresponding authors.

## Author contributions

X-GG and L-PW: conceptualization and writing—original draft. X-GG, L-PW, and GR: data curation. X-GG, GR, and A-YZ: formal analysis. X-GG, L-PW, GR, D-NZ, A-YZ, and CL: methodology. X-GG, A-YZ, and CL: resources. X-GG and GR: software. A-YZ, CL, and D-NZ: supervision. All authors read and agreed to the published version of the manuscript.
