# Supplementary material for: Effects of online mindfulness-based interventions on the mental health of university students: A systematic review and meta-analysis
Source: Front Psychol. 2023 Feb 8;14:1073647. doi: 10.3389/fpsyg.2023.1073647 (PMC9944037; doi:10.3389/fpsyg.2023.1073647)
Supplement: Supplementary file 1 [file Data_Sheet_1.docx]

**Search Strategy**

**Appendix 1. Pubmed**

(“mindful” [Title/Abstract] OR “mindfulness” [Title/Abstract] OR “meditation” [Title/Abstract] OR “MBAT” [Title/Abstract] OR “MBSR” [Title/Abstract] OR “MBCT” [Title/Abstract])

AND

(“online” [Title/Abstract] OR “e-health” [Title/Abstract] OR “Internet” [Title/Abstract] OR “web” [Title/Abstract] OR “computer” [Title/Abstract] OR “cellphone” [Title/Abstract] OR “handset” [Title/Abstract] OR “App” [Title/Abstract] OR “live virtual” [Title/Abstract] OR “VR” [Title/Abstract])

AND

(“student” [Title/Abstract] OR “students” [Title/Abstract])

**Limiters**

- Article type: Randomized Controlled Trial

**Appendix 2. Cochrane library**

Title Abstract Keyword = “mindful” OR “mindfulness” OR “meditation” OR “MBAT” OR “MBSR” OR “MBCT”

AND

Title Abstract Keyword = “online” OR “e-health” OR “Internet” OR “web” OR “computer” OR “cellphone” OR “handset” OR “App” OR “live virtual” OR “VR”

AND//

Title Abstract Keyword = “student” OR “students”

**Limiters**

- Content type: Trials

**Appendix 3. Embase**

All Fields = “mindful” OR “mindfulness” OR “meditation” OR “MBAT” OR “MBSR” OR “MBCT”

AND

All Fields =“online” OR “e-health” OR “Internet” OR “web” OR “computer” OR “cellphone” OR “handset” OR “App” OR “live virtual” OR “VR”

AND

All Fields = “student” OR “students”

**Limiters**

- Article type: Randomized Controlled Trial

**Appendix 4. Web of Science**

All Fields = “mindful” OR “mindfulness” OR “meditation” OR “MBAT” OR “MBSR” OR “MBCT”

AND

All Fields =“online” OR “e-health” OR “Internet” OR “web” OR “computer” OR “cellphone” OR “handset” OR “App” OR “live virtual” OR “VR”

All Fields = “student” OR “students”

**Limiters**

- Document Types: Clinical Trials
- •Document Types: Article
- Languages: English

**Appendix 5. Clinical Trials. gov**

Other terms = “mindful” OR “mindfulness” OR “meditation” OR “MBAT” OR “MBSR” OR “MBCT”

**Limiters**

- Study type: All Studies
- Study Results: Studies with Results
